# Retraction: MHF: A multi-task hybrid fusion method for underwater image enhancement based on biological vision

**DOI:** 10.1371/journal.pone.0360486

**Published:** 2026-10-26

**Authors:** The PLOS One Editors

After this article [1] was published, similarities were noted between this article and previously published work by different authors [2,3, retracted in 4,5, retracted in 6], which call into question the validity and provenance of the reported results. In light of these issues, the *PLOS One* Editors retract this article [1].

The corresponding author responded to the concerns regarding similarity with [2]. The editors do not consider the concerns resolved.

All authors did not agree with the retraction.
